# Standing on single foot-binding test yields satisfactory results as a novel method for the diagnosis of distal tibiofibular syndesmosis instability: a prospective, cross-sectional diagnostic-accuracy study

**DOI:** 10.1186/s12891-023-07155-6

**Published:** 2024-01-12

**Authors:** Shouqi Sun, Tianshi Tang, Pengtao Shi, Chen Yang, Wenjuan Wang, Lei Chen, Min Wei

**Affiliations:** 1 Medical School of Chinese PLA, (BEIJING, Chinese PLA General Hospital, Beijing, China; 2 Department of Orthopedics, the Fourth Medical Center, Chinese PLA General Hospital (BEIJING, Beijing, China; 3 Department of Orthopedics/Chinese National Clinical Research Center for Orthopedics, Sports Medicine and Rehabilitation (BEIJING, Chinese PLA General Hospital, Beijing, China

**Keywords:** Arthroscopy, Cross-sectional, Diagnostic-accuracy study, Distal tibiofibular syndesmosis instability (DTSI), Prospective, Standing on single foot-binding test

## Abstract

**Background:**

Non-invasive diagnosis of distal tibiofibular syndesmosis instability (DTSI) was a great challenge to clinicians. We designed a new method, the Standing on single foot-Binding test, and investigated the accuracy of the test in the diagnosis of distal tibiofibular syndesmosis instability in adults with a history of ankle injury.

**Methods:**

85 participants with ankle injury were subjected to the Standing on single foot-Binding test, MRI and palpation to detect the distal tibiofibular syndesmosis instability (DTSI) and the findings were compared with ankle arthroscopic results. Both participants and arthroscopist were blind to the predicted results of the clinical tests. Sensitivity, specificity, PPV, NPV, LR+, LR − and their 95% CIs were calculated for each of the clinical tests as well as for the positive clinical diagnosis.

**Results:**

The Standing on single foot-Binding test (SOSF-B test) outperformed MRI and palpation, in terms of sensitivity (87.5%/84.38%), specificity (86.79%/86.79%), PPV (80%/79.41%), NPV (92%/91.2%), LR+ (6.625/6.39), LR- (0.14/0.18) and diagnostic accuracy (87.06/85.88), among others, in the diagnosis of distal tibiofibular syndesmosis instability (DTSI). The diagnostic performance of 20° SOSF-B test was virtually identical to that of 0° SOSF-B test. According to the prevalence (28.7%) of DTSI and LR of four tests, the post-test probability could be used in clinical practice for the prediction of DTSI.

**Conclusion:**

This prospective and double-blind diagnostic test showed that the SOSF-B test is clinically feasible for the diagnosis of distal tibiofibular syndesmosis instability (DTSI), and new diagnostic tools for rapid screening of distal tibiofibular syndesmosis instability (DTSI).

**Level of evidence:**

II.

## Background

Distal tibiofibular syndesmosis injury, known as the high ankle sprain, represents a common complication of the ankle injury. About 18% of ankle sprains and 23% of the ankle joint fracture have concomitant distal tibiofibular syndesmosis injury [28]. Distal tibiofibular syndesmosis includes the anterior inferior tibiofibular ligament (AITFL), posterior inferior tibiofibular ligament (PITFL), tibiofibular interosseous ligament (TFIL), and transverse tibiofibular ligament (TTFL) and plays a crucial part in maintaining the stability of the ankle joint [17].

Distal tibiofibular syndesmosis injury, on the basis of MRI findings and patients’ motion state, falls into three types. With type I, or strain type, MRI shows the injury of the distal tibiofibular syndesmosis but the patient has no problems walking; With type II (instability type), MRI exhibits the distal tibiofibular syndesmosis injury and the patient has trouble walking, due to susceptibility to twisting and ankle pain; With type III type (also referred to as separation type), MRI reveals complete rupture of the distal tibiofibular syndesmosis, frequently accompanied with fracture. Type II and type III usually require surgical treatment [16, 26]. Type II (instability type) is much more difficult to diagnose since only ligament is injured and ankle symptoms are atypical. In view of this, we designate type II (instability type) as distal tibiofibular syndesmosis instability (DTSI).

We statistically analyzed the 293 patients who had a history of ankle sprain without fracture and received ankle arthroscopic exploration including checkup of distal tibiofibular space, between October 2017 and December 2020, from the Chinese PLA general hospital. The result showed that 84 cases had DTSI, with an incidence of 28.7%. Therefore, we were led to believe that DTSI is an important subtype of the high ankle injury, and improving the diagnostic accuracy is clinically of great significance. Up till now, there are no generally-accepted diagnostic criteria for DTSI and well-established indications for surgery.

The patients with DTSI usually suffer from ankle pain, discomfort and easy sprain, but these symptoms are not specific [10, 27]. The biomechanical study showed that the distal tibiofibular joint is highly stable [27]. Type II distal tibiofibular syndesmosis injury (distal tibiofibular syndesmosis instability, DTSI) is easy to be missed in diagnosis because it presents no conspicuous signs of fracture and separation. Among the radiographic tools, MRI has been found to possess good specificity and sensitivity in the diagnosis of syndesmosis injury. Nonetheless, MRI remains relatively costly [19]. A meta-analysis involving multiple studies on the examination methods for distal tibiofibular syndesmosis injury evaluated the accuracy of multiple clinical tests and found that no single clinical trial could convincingly predict the disease [18]. The physical examination methods currently used in clinical practice include squeeze test, dorsiflexion-compression test, dorsiflexion-external rotation test, manual stability test, crossed-leg test, heel thump test [10]. Nonetheless, these techniques mainly focus on distal tibiofibular syndesmosis injury and can’t judge the degree of injury [10]. These tests diagnose distal tibiofibular syndesmosis injury by inducing pain and the squeeze test is the only test that yields a clinically relevant result [18]. Ankle arthroscopy is the gold standard for the diagnosis of ankle syndesmosis injury, but in clinical practice, caution should be exercised since ankle arthroscopy is an invasive procedure under anesthesia [4, 7, 24, 25].

So far, non-invasive diagnosis of DTSI has been a great challenge to clinicians. At present, elastic fixation for the DTSI can achieve good surgical results [8]. Therefore, we assume that a similar effect can be obtained by strengthening the distal tibiofibular joint with an external device. In this study, we developed a new strategy for diagnosing DTSI: Standing on single foot-Binding test (SOSF-B test) and investigated the accuracy of the test in the diagnosis of DTSI in adults with a history of ankle injury.

## Methods

This single-center study was approved by the Ethics Committee of Chinese PLA General Hospital (2021 − 637).

### Participant selection

We selected patients from the orthopaedic department from January 1, 2021 to January 31, 2022 according to the established criteria. Included in the study were the candidates satisfying the following criteria: (1) patients suffering from pain or instability due to ankle sprain; (2) patients scheduled for an ankle arthroscopic procedure. The exclusion criteria included: (1) candidates with ankle deformity; (2) those who had previously received ankle surgery; (3) those incapable of standing up; (4) those who eventually refused to receive the surgery; (5) those whose distal tibiofibular joint space had not been arthroscopically explored (joint arthrodesis).

Ethical approval was obtained from the Ethics Committee of Chinese PLA General Hospital (2021 − 637).

### Binding device and measurement equipment

**Fig. 1:**
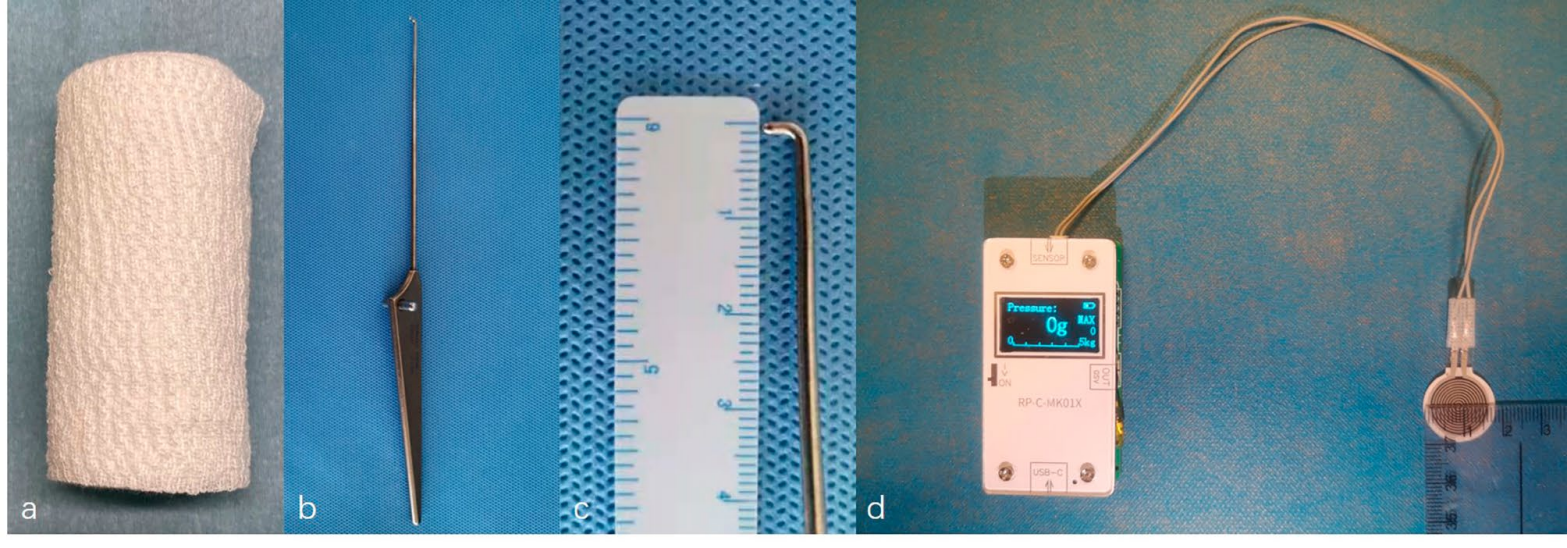
Elastic bandage **(a)**, arthroscopic probe **(b and c)** and pressure index instrument **(d)**

On the basis of our previous experience, normal blood pressure of human ankle superficial vein and pre-experimental results, we elected to employ elastic bandage (TIANJIN TENAI NEW MEDICAL SUPPLIES&TEXTILE TECHNIQUE CO., LTD) (Fig. 1a) to tightly bind the patients’ tibiofibular joint in non-weight-bearing position, with pressure index set at 50 ~ 60 g (*The pressure was determined by pre- experiment*).

An arthroscopic probe (Smith & Nephew) was used for measurement and the diameter was1mm (Fig. 1b and c). The pressure index instrument consisted of a standard pressure sensor and an electronic display module (Fig. 1d).

### Binding method

#### The standing on single foot test

**Fig. 2:**
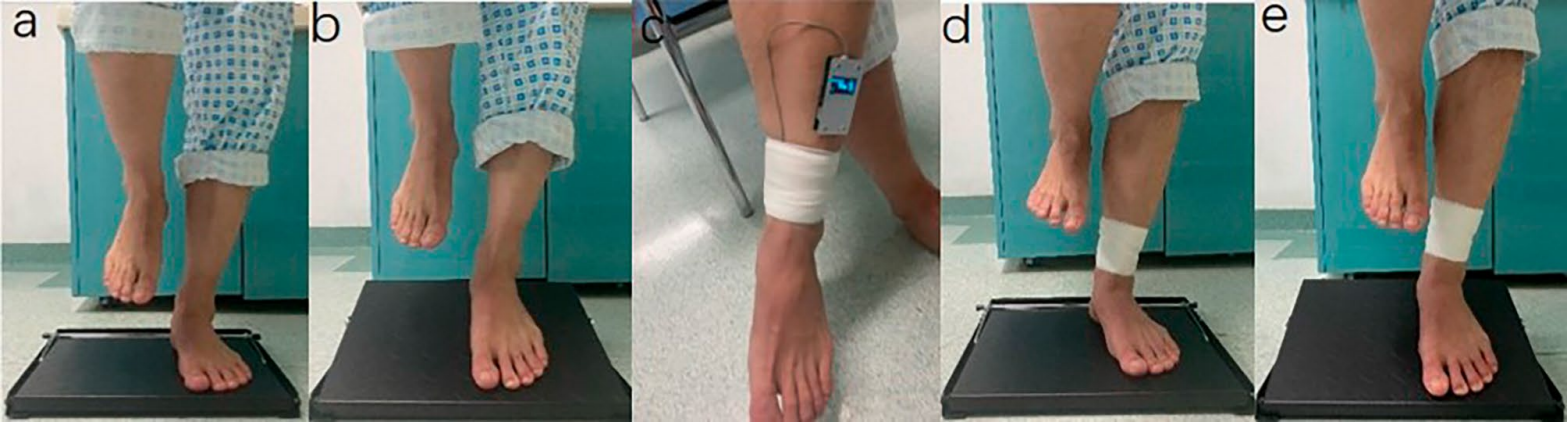
SOSF-B test. The patient stands on the affected foot on the 0° flat **(a)** and 20 ° flat **(b)**; The elastic bandage is tightly wrapped around the affected ankle in non-weight-bearing position **(c)**; The patient stands on the affected foot wrapped with elastic bandage on the 0° flat **(d)** and 20 ° flat **(e)**

The patient was instructed to stand on single foot, with upper limbs dropping naturally, on the 0° flat and 20 ° flat for 5 s (Fig. 2a and b) (*The Standing time was determined by pre- experiment*). By comparing the standing state on both sides, standing on single foot test was considered positive if (1) the patient was unable to stand on single foot for 5 s; or (2) the patient could stand on single foot for 5 s, but had two of the following conditions: (a) The patient’s upper body shook obviously; (b) The patient had to adjust the foot position to complete the standing; (c) The patient reported that the contraction of the posterior leg muscle group was more obvious on the affected side than on the healthy side.

#### The standing on single foot-binding test

According to the pressure setting, the elastic bandage was tightly wrapped around the affected ankle (Fig. 2c). The standing on single foot test was repeated (Fig. 2d and e). The situations of standing before and after binding were compared, and the binding test was considered positive if the positive sign of the affected side disappeared or weakened.

#### Standing on 0° and 20 ° flat plate

The standing state of patients on 0° flat and 20 ° flat plate were recorded respectively. The physical examination was carried out by the same examiner.

### Syndesmosis ligament palpation and MRI

Syndesmosis ligament palpation and MRI were used for checking ankle syndesmosis injury. Syndesmosis ligament palpation included the tenderness at projection point of body surface of AITFL/PITFL-transverse ligament [14]. MRI were evaluated for the presence of syndesmotic injury [9, 11]. Syndesmosis ligament palpation was performed by the same examiner and the MRI by two experienced radiologist who was blind to the results of clinical tests and arthroscopic exploration.

### Surgical technique

**Fig. 3:**
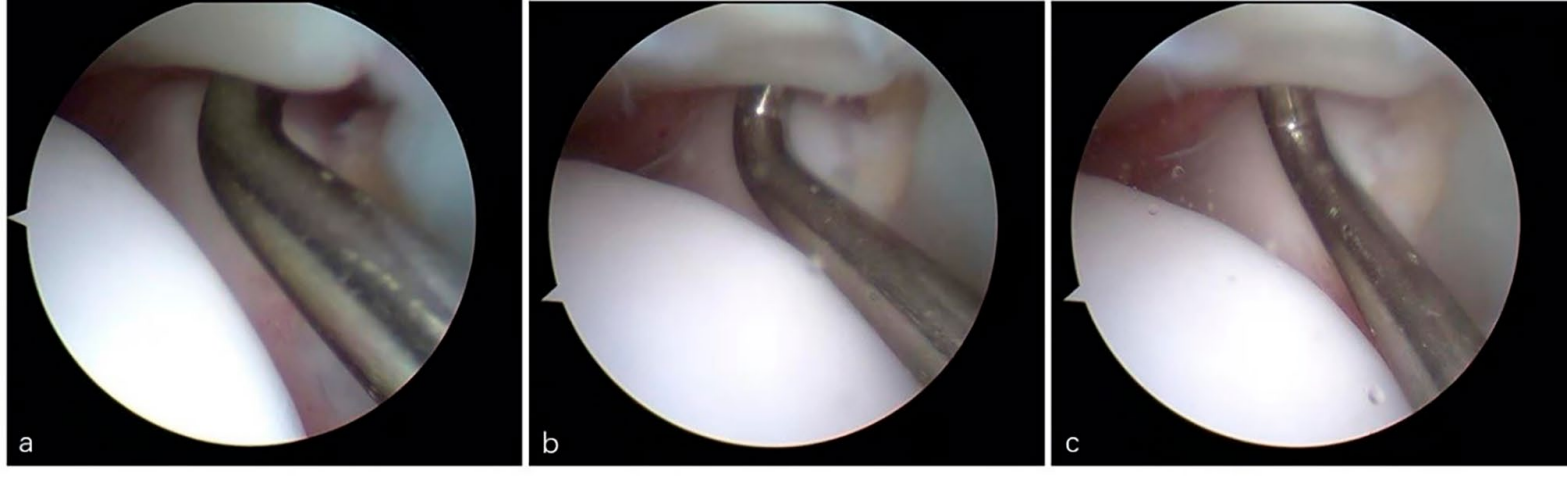
Arthroscopic exploration process. Make the probe tip insert the distal tibiofibular joint space **(a)**; The probe tip opens distal tibiofibular joint space **(b and c)**

The distal tibiofibular syndesmosis stability was arthroscopically probed by checking the distal tibiofibular joint gap. Briefly, the probe tip was inserted into the distal tibiofibular joint space (Fig. 3a), with the hook being rotated axially. If the probe tip could open the distal tibiofibular joint gap (Fig. 3b and c), the distal tibiofibular joint space was greater than 1 mm.

The operation was done by a sports medicine doctor with 30 years of clinical experience. All surgical procedures were photographed and video-recorded.

### Statistical analysis

The statistical analysis was performed by using Statistical Package for Social Sciences software, version 26.0 (SPSS). Measurement data were expressed as mean ± standard deviation (SD) and were rounded to two decimal places. The independent sample *t* test was used for measurement data and chi-square test was employed for enumeration data. Alpha (α) value was set at 0.05.

In order to determine the diagnostic utility of the clinical tests, we compared the physical examination results with the arthroscopic diagnosis. A series of 2 × 2 contingency tables were generated, using the arthroscopic diagnosis (positive or negative for DTSI) as the reference standard. Sensitivity, specificity, PPV (positive predictive value), NPV (negative predictive value), LR+ (positive likelihood ratio), LR− (negative likelihood ratio) and their 95% CIs were calculated for each of the clinical tests as well as for the positive clinical diagnosis [6, 12, 19]. The diagnostic accuracy was calculated as: (True positive + True negative)/Total number of cases [13].

In this study, prevalence represented the pre-test probability of a particular diagnosis in all listed cases. Post-test probability allows for estimation of how much the examiner’s findings influenced the accuracy of the diagnosis when the test yielded a negative or a positive result.

Sample size was estimated by PASS 11 and met the statistical requirements.

### Patient and public involvement

It was not appropriate or possible to involve patients or the public in the design, or conduct, or reporting, or dissemination plans of our research.

## Results

**Fig. 4:**
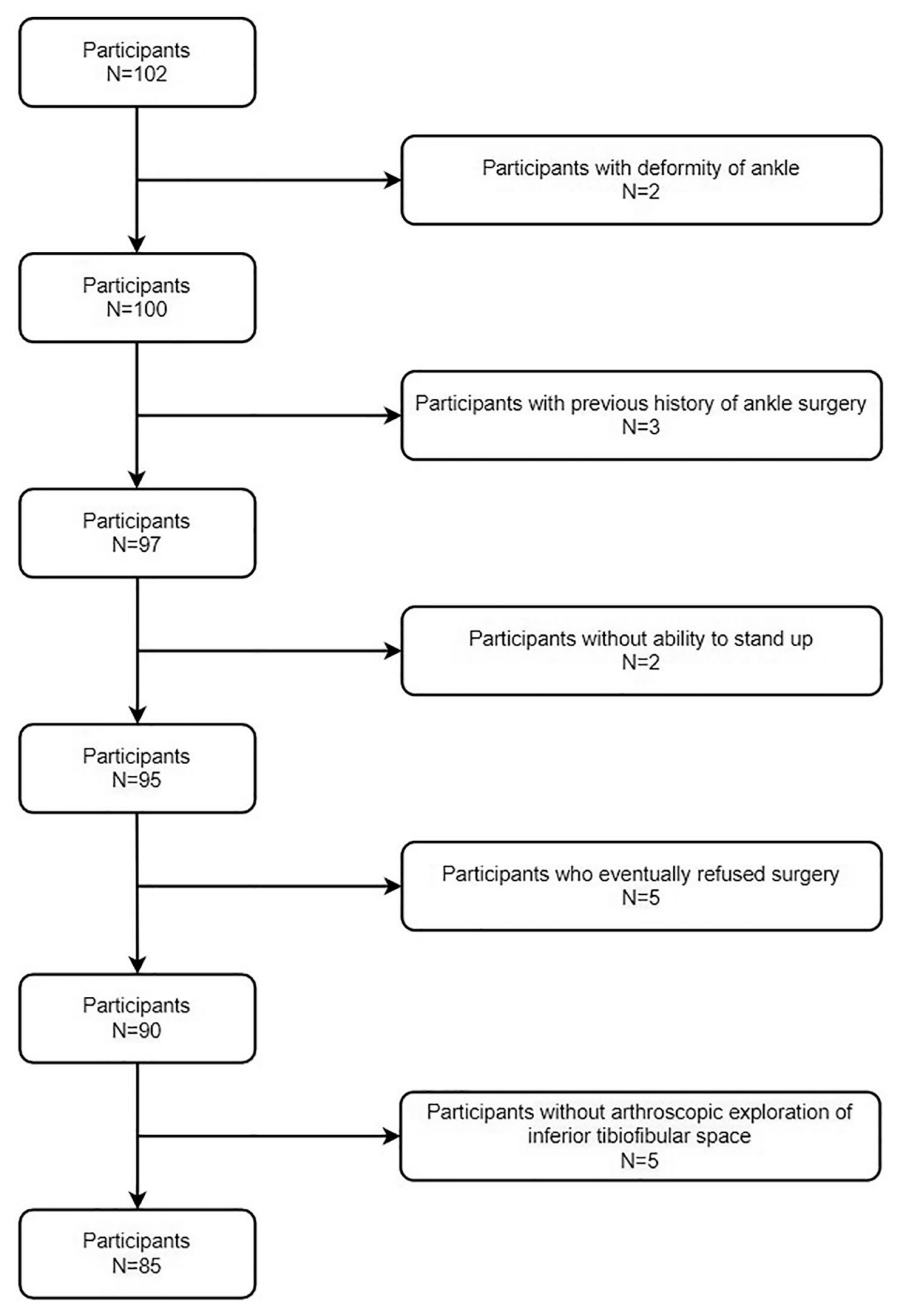
Flow chart of participant recruitment

There were 85 cases in our cohort, and all participants had a definite history of ankle joint trauma. In terms of the results of ankle arthroscopic exploration, the participants were divided into two groups. Figure 4 outlines the exclusion and inclusion of participants throughout the study. Participants’ characteristics, including age, gender, laterality, height, weight and BMI, are given in Table 1.

**Table 1:** General features of the participants

Item	Total (N = 85)	With DTSI (N = 32)	Without DTSI (N = 53)
Age(year)	33.77 ± 11.09, (18 ~ 64)	29.91 ± 9.94, (17 ~ 55) #	36.38 ± 11.2, (19 ~ 64) **#**
Gender(male: female)	59:26	18:14*	40:13*
Side(left: right)	37:48	17:15#	26:27#
Height (m)	1.73 ± 0.08, (1.55 ~ 1.87)	1.72 ± 0.76, (1.55 ~ 1.84) *	1.74 ± 0.08, (1.58 ~ 1.87) *
Weight (Kg)	76.04 ± 12.55, (52 ~ 115)	74.05 ± 12.39, (52 ~ 95) *	77.4 ± 12.06, (53 ~ 115) *
BMI (kg/m²)	25.38 ± 3.43, (18.87 ~ 34.06)	25.13 ± 3.94, (18.87 ~ 34.06) *	25.55 ± 3.07, (19.83 ~ 33.97) *

(mean ± SD). #: *P* < 0.05; *: *P* > 0.05

Ankle arthroscopy revealed that there were three main complications in patients with DTSI: synovitis, osteochondral lesion of the talus and chronic lateral ankle syndesmosis injury (Table 2).

**Table 2:** Complications of ankle injury using arthroscopic findings as the reference standard

Complications	Total (N = 85)	With DTSI (N = 32)	Without DTSI (N = 53)
A	11(13%)	5(16%)	6(11%)
A + B	14(17%)	2(6%)	12(23%)
A + C	47(55%)	22(69%)	25(47%)
A + B + C	13(15%)	3(9%)	10(19%)

A: synovitis; B: osteochondral lesion of the talus; C: chronic lateral ankle syndesmosis injury

Overall, the diagnostic accuracy of 20° SOSF-B test (87.06%) and 0° SOSF-B test (85.88%) was virtually identical and was significantly better than that of MRI (72.94%) and syndesmosis ligament palpation (67.06%). In addition, the 20° SOSF-B test (87.5%) and 0° SOSF-B test (84.38%) had the highest sensitivity and same specificity (86.79%). The 20° SOSF-B test (80%) and 0° SOSF-B test (79.41%) had the higher PPV compared with MRI (60.98%) and syndesmosis ligament palpation (54.76%). What is more, the 20° SOSF-B test (92%) and 0° SOSF-B test (91.2%) had the higher NPV in comparison with MRI (84.09%) and syndesmosis ligament palpation (79.07%). Furthermore, LR + and LR- of the four clinical diagnostic methods were of value in clinical practice. The LR + for 20° SOSF-B test (6.625) and 0° SOSF-B test (6.39) indicated a moderate increase in the likelihood of the disease if the test result was positive. The LR + for MRI (2.59) and syndesmosis ligament palpation (2.01) suggested a small increase in the likelihood of the disease if the test result was positive. The LR − for the 20° SOSF-B test (0.14) and 0° SOSF-B test (0.18) was indicative of a moderate decrease in the likelihood of the disease with if the test result was negative. The LR − for MRI (0.31) and syndesmosis ligament palpation (0.44) was suggestive of a small decrease in the likelihood of the disease if the test result was negative (Table 3).

**Table 3:** Clinical test results and indices of diagnostic utility in the diagnosis of DTSI using arthroscopic findings as the reference standard

TEST		Arthroscopic diagnosis + -		Sensitivity (%) (95% CI)	Specificity (%) (95% CI)	PPV (%) (95% CI)	NPV (%) (95% CI)	LR+ (95% CI)	LR- (95% CI)	Diagnostic accuracy (%)
20° SOSF-B test	**+**	28	7	87.5 (70.07–95.92)	86.79 (74.05–94.09)	80 (62.54–90.94)	92 (79.89–97.41)	6.625 (3.28–13.37)	0.14 (0.06–0.36)	87.06
	**-**	4	46							
0° SOSF-B test	**+**	27	7	84.38 (66.45–94.10)	86.79 (74.05–94.09)	79.41 (61.59–90.66)	91.2 (77.81–96.33)	6.39 (3.15–12.94)	0.18 (0.08–0.41)	85.88
	**-**	5	46							
MRI	**+**	25	16	78.13 (59.56–90.06)	69.81 (55.49–81.26)	60.98 (44.54–75.38)	84.09 (69.33–92.84)	2.59 (1.65–4.05)	0.31 (0.16–0.61)	72.94
	**-**	7	37							
Palpation	**+**	23	19	71.88	64.15	54.76	79.07	2.01	0.44	67.06
	**-**	9	34	(53.02–85.60)	(49.75–76.51)	(38.83–69.83)	(63.52–89.42)	(1.32–3.05)	(0.25–0.78)	

Statistically significant (*P* < 0.05). PPV, positive predictive value; NPV, negative predictive value; LR+, positive likelihood ratio; LR−, negative likelihood ratio

**Fig. 5:**
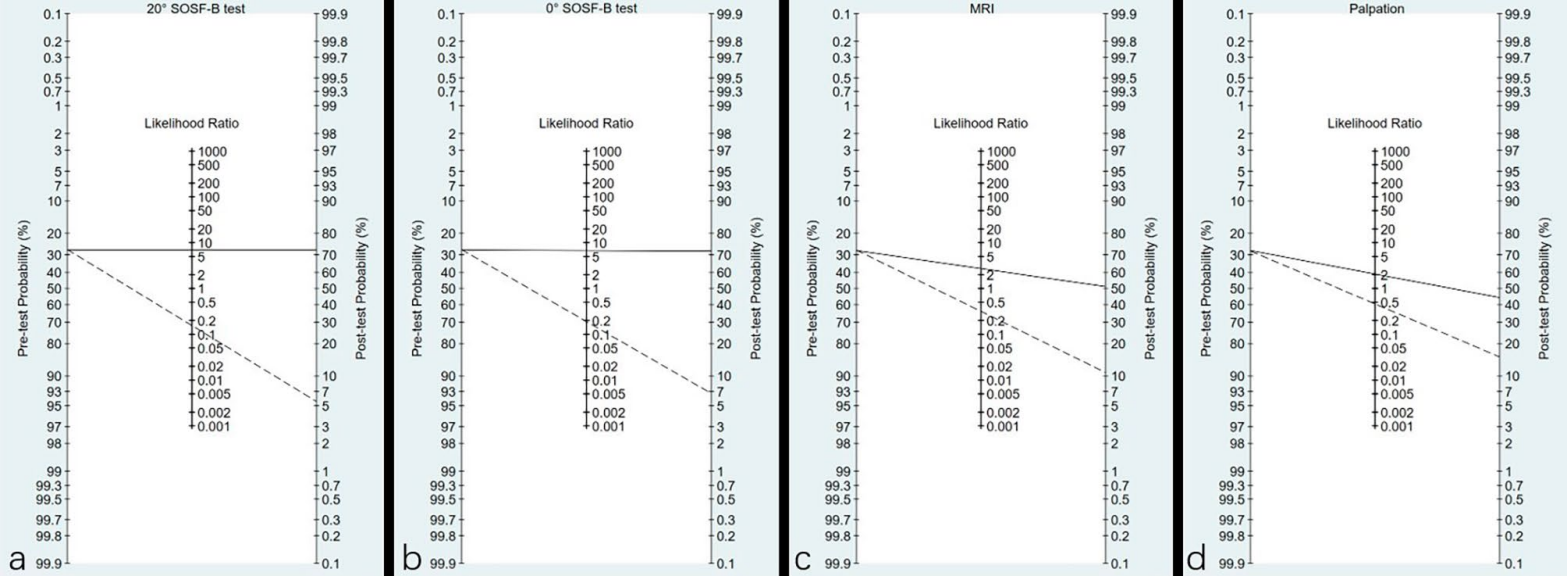
The Fagan nomogram predicting post-test probability from prevalence and LR. 20° SOSF-B test **(a)**, 0° SOSF-B test **(b)**, MRI **(c)** and palpation **(d)** respectively represent. The solid line is based on LR (+), and the dash line is based on LR (-)

This continuous nature of LR and their implication in shifting probabilities when the test result is positive or negative can be graphically illustrated on a Fagan nomogram, which could also be clinically used. According to the prevalence (28.7%) of DTSI and LR + of the four tests, the post-test probability of 20° SOSF-B test, 0° SOSF-B test, MRI and palpation was 72.7%, 72%, 51% and 44.7%. According to the prevalence (28.7%) of DTSI and LR- of the four tests, the post-test probability of 20° SOSF-B test, 0° SOSF-B test, MRI and palpation was 5.3%, 6.8%, 11.1% and 15%, respectively (Fig. 5).

## Discussion

Our research team, from clinical and biomechanical perspectives, designed the Standing on Single Foot-Binding test (SOSF-B test), which used an external bandage to partially replace the function of the distal tibiofibular syndesmosis, to diagnose DTSI. Our analysis of the diagnostic performance (Table 3) exhibited that the test had good sensitivity and specificity in detecting DTSI, as compared with palpation and MRI. Han SH believes that MRI performs well in the diagnosis of ligament injury, but it is unable to quantitatively determine the degree of joint laxity by dynamic inspection [23]. This study proved that MRI had poor specificity (69.81%) in the diagnosis of DTSI when compared with 0° SOSF-B test (86.79%). The 20 ° SOSF-B test (87.5%) showed marginally better sensitivity than 0° SOSF-B test (84.38%), but it requires a special device and had the same specificity as that of 0° SOSF-B test (86.79%). The post-test probability of 0° SOSF-B test, derived from the prevalence (28.7%) and LR (+) was 72%, suggesting that the probability of DTSI for a person in this hypothetical population increases from 28.7 to 72% when he or she has a positive result with the 0° SOSF-B test. The post-test probability of 0° SOSF-B test was 6.8%, derived from the prevalence (28.7%) and LR (-), indicating that when the 0° SOSF-B test yields a negative result, a person’s chance of having DTSI drops from 28.7 to 6.8% in this population [3]. We believe that the 0° SOSF-B test can satisfy the needs of clinical diagnosis and can serve as a new alternative of physical examinations for diagnosing DTSI.

Our aim is to seek a new tool for rapid outpatient screening of DTSI, and we are pleasantly surprised to find that the SOSF-B test can better diagnose diseases compared with MRI and palpation. This may be because the SOSF-B test is similar to a disease treatment method, that ankle joint binding is often used as a treatment option for ankle instability in clinical practice and may have good therapeutic effects [1], and overcomes the disadvantage that other diagnostic methods cannot dynamically observe the patient’s movement state. This test is characterized by easy operation, relative objectivity of indicators, a higher acceptance and a short learning curve. The standing state of patients with one foot is relatively objective, and the false positive rate is low. The best part of the method is that when checking the patient’s condition during exercise, an external elastic bandage functionally replaces the distal tibiofibular syndesmosis. And patients with DTSI are more willing to cooperate with the examiner since pain or instability are relieved, and, as a result, the diagnostic accuracy is improved. In general, when the patient reports a recent injury, and complains of inability to walk following injury and physical checkup reveals tenderness, the SOSF-B test is highly recommended to confirm or eliminate the diagnosis. When the SOSF-B test is positive, DTSI is highly likely and confirmatory imaging should ensue. But we also need to note that long term SOSF-B test can cause obstruction of blood flow in the lower limbs of patients and adverse symptoms such as numbness and pain. Therefore, the examination process should not be too long and the patient’s condition should be observed at any time.

The selection of pressure index and standing time in this test all come from the pre-experiment. (1) We set 50 ~ 60 g as the pressure index, because the binding effect was the best under this condition. The lower pressure index couldn’t play the role of compression, while the patients with the higher pressure felt obvious discomfort in the lower limbs, which might be related to the fact that too strong pressure will block the blood circulation at the ankle; (2) We used 5 s as the standing time basing on the results of the pre-experiment. In the pre-experiment, we used 1 s, 5 and 10 s to detect the impact of the standing time. It found that 1 s was so short that the observer and patient did not have enough time to judge the standing state. And some patients felt increasing pain on the affected side, which affected their self-evaluation, when standing for 10 s.

According to the results of arthroscopic exploration, the research group found DTSI could be accompanied by a variety of complications such as synovitis, osteochondral lesion of the talus and chronic lateral ankle syndesmosis injury [2, 5, 22]. The results of arthroscopic exploration shew that the prevalence of DTSI exceeded 37% (32/85) and 16% (5/32) of DTSI patients were accompanied by only synovial hyperplasia. The SOSF-B test designed by the research group was only aimed at how to accurately diagnose DTSI and ignored other complications of ankle sprain, which highlighted the significance of the SOSF-B test in the diagnosis of special disease.

This study has some limitations. First, the participants were selected from patients who have been found to have dyskinesia of ankle joint in the outpatient department and were recommended or scheduled for surgery. Therefore, caution should be exercised when the results are extrapolated to healthy people. Second, EMG (electromyogram) may be a better objective index to judge the muscle contraction of patients, but because it is an invasive examination, it is not suitable to be widely carried out in sports medicine clinics [21]. Third, the test may be substantially affected by the presence of generalized ligament laxity [15, 20]. In our cohort, 9 cases had multiple generalized ligament laxity and 3 cases were misdiagnosed. How generalized ligament laxity affects the stability of distal tibiofibular syndesmosis warrants further studies. Fourth, according to statistical data (Table 1), age might be another important contributor to DTSI and it might be related to the higher or different sports activities engaged by young people. The correlation between age and DTSI needs to be further studied in future research. Therefore, we recommend that future studies use prediction models that include multiple combinations of clinical symptoms and signs and diagnostic tests. This would help to better investigate the diagnostic power of these tests in the determination the injury severity.

To sum up, the SOSF-B test has satisfactory sensitivity and specificity and can serve as an excellent alternative of physical examinations, though the symptoms of patients with ankle injury vary. We believe that the result of the test is clinically helpful in the diagnosis of DTSI patients.

## Conclusion

The prospective and double-blind diagnostic-accuracy study demonstrated that the Standing on single foot-Binding test (SOSF-B test) could be used as a new clinical diagnostic experiment for diagnosing distal tibiofibular syndesmosis instability (DTSI), and may play a role in the diagnosis and treatment of ankle sprain.

## Data Availability

All data are available upon reasonable request from the corresponding author.

## Abbreviations

AITFL: anterior inferior tibiofibular ligament
PITFL: posterior inferior tibiofibular ligament
TFIL: tibiofibular interosseous ligament
TTFL: transverse tibiofibular ligament
DTSI: distal tibiofibular syndesmosis instability
SOSF-B test: Standing on single foot-Binding test
PPV: positive predictive value
NPV: (negative predictive value
LR+: positive likelihood ratio
LR−: negative likelihood ratio
EMG: electromyogram

## References

[1] Biz C, Nicoletti P, Tomasin M (2022). Is Kinesio Taping Effective for Sport performance and ankle function of athletes with chronic ankle instability (CAI)? A systematic review and Meta-analysis. Med (Kaunas).

[2] Blázquez Martín T, Iglesias Durán E, San Miguel Campos M (2016). Complications after ankle and hindfoot arthroscopy. Rev Esp Cir Ortop Traumatol.

[3] Bolboacă SD. Medical diagnostic tests: a review of test anatomy, phases, and statistical treatment of data. Comput Math Methods Med,2019;28;2019:1891569. 10.1155/2019/1891569 PMC6558629 31275427

[4] Chan KB, Lui TH (2016). Role of Ankle Arthroscopy in Management of Acute Ankle fracture. Arthroscopy.

[5] Chun K-Y, Choi YS, Lee SH (2015). Deltoid ligament and Tibiofibular Syndesmosis Injury in chronic lateral ankle instability: magnetic resonance imaging evaluation at 3T and comparison with arthroscopy. Korean J Radiol.

[6] Cleland J, Koppenhaver S, Su J. The reliability and diagnostic utility of the orthopaedic clinical examination. In: Cleland J, Koppenhaver S, Netter F, edsNetter’s orthopaedic clinical examination: an evidence-based approach. Elsevier Health Sciences. 2015:1–21.

[7] Dalmau-Pastor M, El-Daou H, Stephen JM (2023). Clinical relevance and function of Anterior Talofibular Ligament Superior and Inferior fascicles: a robotic study. Am J Sports Med.

[8] Gohar AN 1, Cunningham P, Lynch B et al. Fixation of ankle syndesmotic injuries: comparison of tightrope fixation and syndesmotic screw fixation for accuracy of syndesmotic reduction. Am J Sports Me, 2012;40(12):2828-35. 10.1177/0363546512461480 23051785

[9] Hermans JJ, Wentink N, Beumer A, Hop WCJ, Heijboer MP, Moonen AFCM, Ginai AZ (2012). Correlation between radiological assessment of acute ankle fractures and syndesmotic injury on MRI. Skeletal Radiol.

[10] Jiao C, Gui J, Kurokawa H (2021). APKASS Consensus Statement on Chronic Syndesmosis Injury, Part 1: clinical manifestation, radiologic examination, diagnosis criteria, classification, and Nonoperative Treatment. Orthop J Sports Med.

[11] Langner I, Frank M, Kuehn JP, Hinz P, Ekkernkamp A, Hosten N, Langner S (2011). Acute inversion injury of the ankle without radiological abnormalities: assessment with high-field MR Imagingand correlation of findings with clinical outcome. Skeletal Radiol.

[12] Magarey ME, Jones MA, Cook CE, Hayes MG (2016). Does physiotherapy diagnosis of shoulder pathology compare to arthroscopic findings?. Br J Sports Med.

[13] Mentiplay BF, Perraton LG, Bower KJ (2015). Assessment of Lower Limb muscle strength and power using hand-held and fixed Dynamometry: a reliability and validity study. PLoS ONE.

[14] Mulligan EP (2011). Evaluation and management of ankle syndesmosis injuries. Phys Ther Sport.

[15] Razak HRBA, Ali NB, Howe TS (2014). Generalized ligamentous laxity may be a predisposing factor for musculoskeletal injuries. J Sci Med Sport.

[16] Ryan LP, Hills MC, Chang J, Wilson CD (2014). The lambda sign: a new radiographic indicator of latent syndesmosis instability. Foot Ankle Int.

[17] Sharif B, Welck M, Saifuddin A (2020). MRI of the distal tibiofibular joint. Skeletal Radiol.

[18] Sman AD, Hiller CE, Refshauge KM (2013). Diagnostic accuracy of clinical tests for diagnosis of ankle syndesmosis injury: a systematic review. Br J Sports Med.

[19] Sman AD, Hiller CE, Rae K (2015). Diagnostic accuracy of clinical tests for ankle syndesmosis injury. Br J Sports Med.

[20] Sueyoshi T, Emoto G, Yuasa T (2016). Generalized joint laxity and Ligament Injuries in High School-aged female Volleyball players in Japan. Orthop J Sports Med.

[21] Tankisi H, Burke D, Cui L (2020). Standards of instrumentation of EMG. Clin Neurophysiol.

[22] Theodorakys Marín Fermín JM, Hovsepian P (2021). Arthroscopic debridement of osteochondral lesions of the talus: a systematic review. Foot (Edinb).

[23] Tourné Y, Molinier F, Andrieu M (2019). Diagnosis and treatment of tibiofibular syndesmosis lesions. Orthop Traumatol Surg Res.

[24] Vega J, Peña F, Golanó P (2016). Minor or occult ankle instability as a cause of anterolateral pain after ankle sprain. Knee Surg Sports Traumatol Arthrosc.

[25] Wake J, Martin KD (2020). Syndesmosis Injury from diagnosis to repair: physical examination, diagnosis, and arthroscopic-assisted reduction. J Am Acad Orthop Surg.

[26] Yu GS, Lin YB, Xiong GS (2019). Diagnosis and treatment of ankle syndesmosis injuries with associated interosseous membrane injury: a current concept review. Int Orthop.

[27] Yuen CP, Lui TH (2017). Distal tibiofibular syndesmosis: anatomy, Biomechanics, Injury and Management. Open Orthop J.

[28] Zhang P, Liang Y, He JS (2017). A systematic review of Tightrope versus syndesmotic screw in the treatment of distal tibiofibular syndesmosis injury. BMC Musculoskelet Disord.

